# Preparation and Characterization of a Pectin Membrane-Based Optical pH Sensor for Fish Freshness Monitoring

**DOI:** 10.3390/bios9020060

**Published:** 2019-04-26

**Authors:** Uswatun Hasanah, Mita Setyowati, Rustam Efendi, Muslem Muslem, Nor Diyana Md Sani, Eka Safitri, Lee Yook Heng, Rinaldi Idroes

**Affiliations:** 1 Graduate School of Mathematics and Applied Sciences, Universitas Syiah Kuala, Banda Aceh 23111, Indonesia; uswatun.hasanah@utu.ac.id; 2 Department of Fisheries, Faculty of Fisheries and Marine Sciences, Universitas Teuku Umar, West Aceh 23615, Indonesia; 3 Department of Agronomy, Faculty of Agriculture, Universitas Teuku Umar, West Aceh 23615, Indonesia; mitasetyowati@utu.ac.id; 4 Department of Chemistry, Faculty of Mathematics and Natural Sciences, Universitas Syiah Kuala, Banda Aceh 23111, Indonesia; rustamefendi706@gmail.com (R.E.); moslem_coolam@yahoo.com (M.M.); e.safitri@yahoo.co.id (E.S.); 5 Sanichem Resources Sdn. Bhd. No 7 & 7A Jalan Timur 6/1A Mercato @Enstek, Bandar Estek NSN 71060, Malaysia; diyanasani@yahoo.com; 6 School of Chemical Sciences and Food Technology, Faculty of Science and Technology, Universiti Kebangsaan Malaysia, Bangi SGR 43600 UKM, Malaysia; leeyookheng@yahoo.co.uk; 7 Southeast Asia Disaster Prevention Research Initiative (SEADPRI-UKM), LESTARI, Universiti Kebangsaan Malaysia, Bangi SGR 43600 UKM, Malaysia; 8 Department of Pharmacy, Faculty of Mathematics and Natural Sciences, Universitas Syiah Kuala, Banda Aceh 23111, Indonesia

**Keywords:** optical pH sensor, pectin, chromoionophore, fish freshness

## Abstract

In a simple and instant procedure for detecting fish freshness, a hydrogel and hydrophilic pectin matrix membrane was used successfully as an optical pH sensor by immobilizing the chromoionophore ETH 5294 (CI), which is very selective and sensitive for the membrane. The Pe/CI optical pH sensor exhibited excellent linearity between pH 5 and pH 9, with a sensor response time of 5 min and reproducibility of 1.49% relative standard deviation (RSD). The sensor showed response stability for 15 days and a response reduction of 8.6%. The sensor’s capability was demonstrated by the detection of fish freshness for 17 days at 4 °C.

## 1. Introduction

Fish freshness is a major health concern for consumers. Fish is easily degraded by enzymatic reactions and because of microbial contamination [1,2,3]. Therefore, it is important for the consumer to monitor fish freshness before consumption or industrial processing. The simplest determination method for fish freshness is a physical assessment method where trained panelists gauge a fish’s color, eyes, gills, skin, and meat texture and odor. Data are compiled according to particular degradation schemes to create a quality index (QI) [4]. Nevertheless, this method is inaccurate because of its dependence on the panelists’ abilities and experiences.

A more modern method for determining fish freshness has been developed, which includes laboratory experts and the use of gas chromatography–mass spectrometry instruments. This method determines the concentration of total volatile basic nitrogen (TVB-N), which is formed by the enzymatic decomposition of trimethylamine oxide (TMAO), due to the presence of bacteria in dead fish [5,6,7]. However, this method is not practical for field use, because the procedure must be carried out in a laboratory. Additionally, the said method is destructive, time-consuming, and costly [8]. Fish consumers and, especially, the fishery industry, demand a practical, instant, and non-destructive analysis method [9].

A simpler method for determining fish freshness was developed by using an optical pH sensor. The analysis was conducted by attaching the sensor to the fish meat surface. The TVB-N was determined by the color change of bromocresol, which was immobilized on a polytetrafluoroethylene (PTFE) membrane matrix [10]. This method is superior for food monitoring because it allows a non-destructive and in situ analysis process [11,12]. Nowadays, the development of in situ methods, such as sensor, laser, and electrometric methods, is preferred [13] as there is a shorter analysis time when compared with laboratory methods. However, PTFE is a synthetic material which has a negative impact on the environment. Therefore, we developed a sensor from a biopolymer-based membrane matrix.

One of the biopolymers that can be used as a sensor matrix is pectin. Pectin is a biodegradable anionic polymer, therefore it is environmentally friendly. Pectin is also hydrophilic, allowing higher permeability than hydrophobic synthetic polymers [14]. This can facilitate the adsorption of active substances, allowing a more rapid sensory response. Chromoionophore (CI) is an active substance and a potential pH indicator. CI is selective towards H^+^ [15] and sensitive in applications in either hydrophilic or lipophilic media. CI is also a wide-range pH indicator, which is important for sensor application.

In this research, a hydrogel pectin membrane-based Pe/CI optical pH sensor was developed, where CI was immobilized on the sensor matrix. The changes of pH on fish were detected by CI through the protonation and deprotonation of CI functional groups. The level of protonation and deprotonation at various pHs was calculated by the absorbance, which was determined by a UV–Vis spectrophotometer [16]. The sensor’s performance against variations in buffer pH, response time, lifetime, and sensor reproducibility was then evaluated.

## 2. Experimental

### 2.1. Chemicals and Instruments

Chemicals used in this research were of analytical grade and included chromoionophore I Nile Blue ETH 5294 (CI), monopotassium dihydrogen phosphate (KH_2_PO_4_), and dipotassium hydrogen phosphate (K_2_HPO_4_), purchased from Fluka, and pectin, ethanol (C_2_H_5_OH) absolute, and calcium chloride (CaCl_2_), purchased from Sigma-Aldrich. The absorbance signal from the sensor was determined by a UV–Vis Shimazu 1800 spectrophotometer, and the pH buffer was controlled by a Thermo Orion Star A2111 pH meter.

### 2.2. Preparation of Reagents and Solutions

The CI reagent was prepared by dissolving 0.4 mg of CI in 1 mL of ethanol, followed by stirring for 10 min to obtain a homogenous solution. Potassium phosphate buffer solution (PBS) was prepared by mixing KH_2_PO_4_ with K_2_HPO_4_. A 2% Pectin membrane solution was prepared by dissolving 2 g of pectin in 100 mL of CaCl_2_ 0.1 M (CaCl_2_ 0.1 M was prepared by dissolving it in PBS 0.1 M at pH 7). The membrane solution was heated at 60 °C until it was homogenous.

### 2.3. Preparation of the Pe/CI Optical pH Sensor

**Figure 1:**
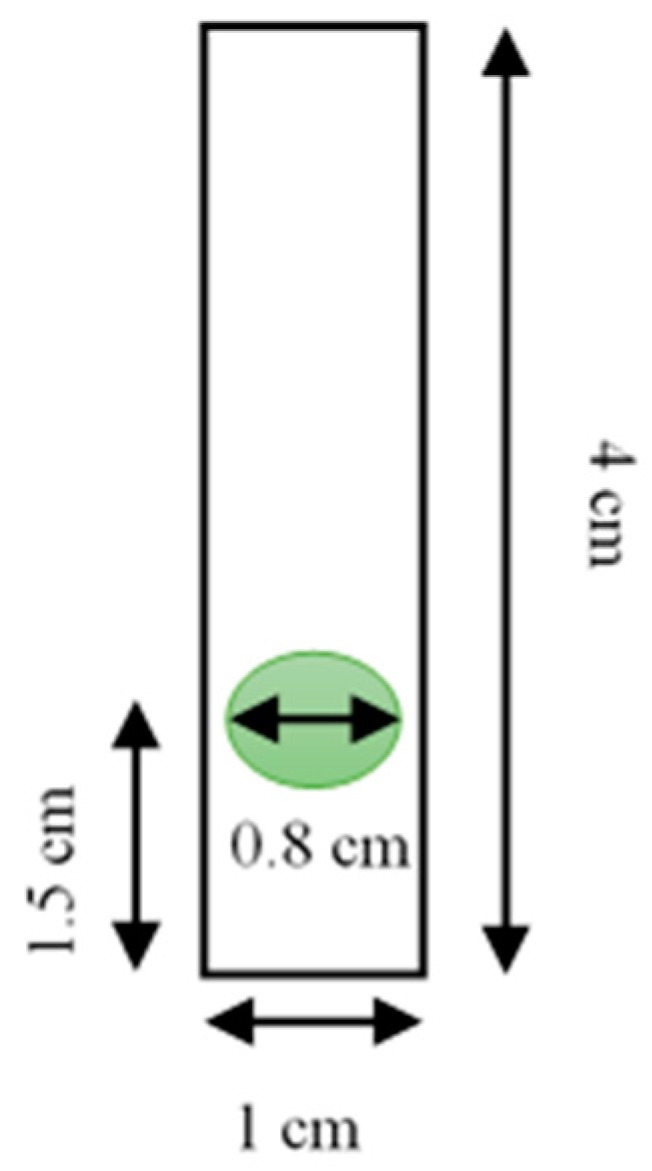
The optical pH sensor plate.

The Pe/CI optical pH sensor was prepared by immobilizing CI within the pectin membrane. First, 1 mL of the 2% pectin membrane solution was poured into 400 μL of CI and stirred slowly for 10 min. As much as 55 μL of the Pe/CI solution was added to an 8 mm-wide plastic slide surface (Figure 1). It was then dried overnight at room temperature (25 °C). The procedure was repeated four times to form a multilayer film.

### 2.4. Response Optimization of the Pe/CI Optical pH Sensor

The performance of the Pe/CI optical pH sensor was tested against variations in pH, response time, lifetime, and reproducibility. The sensor’s response against variations in pH was determined by adding the 0.1 M PBS solution within a pH range of 5–9 to the sensor film. Then, the absorbance was measured at a maximum wavelength

The response time of the sensor was determined by adding the 0.1 M PBS solution at pH 7 to the sensor film and then measuring the absorbance at 535 nm, every minute, for 10 min.

To determine the lifetime or stability of the sensor, 0.1 M PBS solution at pH 7 was added to the sensor film and its absorbance was measured. This procedure was performed each day, for 27 days. The reproducibility of the sensor was determined by the absorbance of UV–Vis at 535 nm from 10 sensors. All determinations were conducted three times for each sensor.

### 2.5. Determination of Fish Freshness

The application of the Pe/CI optical pH sensor was evaluated by determining the pH of a tilapia fish that was stored at 4 °C. The fish sample was removed from the refrigerator and left for a few minutes. The pH measurement was performed by putting the sensor on the surface of fish for 5 min and measuring the absorbance. The pH determination was conducted once every day until the 17th day. As the sensor is highly sensitive to light and temperature, the measurements were performed in the lee (shadow) to minimize exposure to light and heat. After being used, the sensor was stored in the dark at 4 °C.

## 3. Results and Discussion

### 3.1. Absorbance Response of the Pe/CI Optical pH Sensor

**Figure 2:**
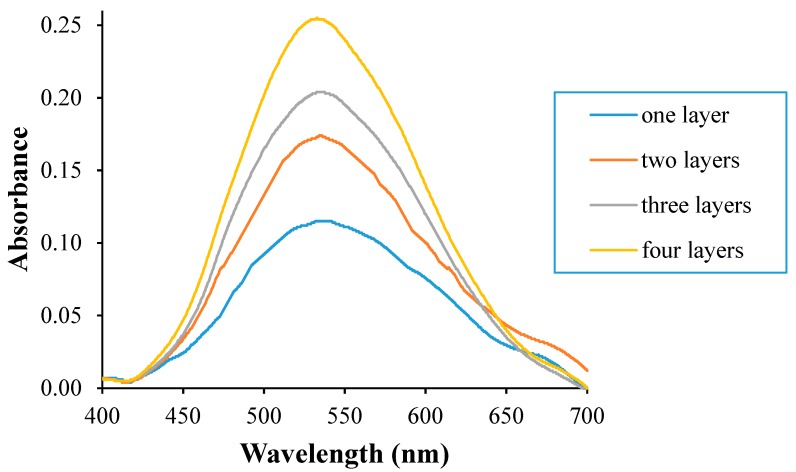
UV–Vis spectra of each pectin/chromoionophore (Pe/CI) optical pH sensor layer. An increasing number of layers increases absorbance.

Pectin is a polymer which is used as a matrix to immobilize CI by entrapment. Pectin is a bio polyelectrolyte which undergoes chain association and forms a hydrogel after the addition of CaCl_2_, which contains divalent cations (Ca^2+^). When CI is immobilized on pectin, CI is trapped in the polymeric membrane of pectin, forming a film. This film will change from purple to blue when CI is protonated and from blue to pink when CI is deprotonated. The change of film color depends on the environmental pH. This behavior is the basic reason why Pe/CI was used as the indicator for pH changes. The Pe/CI optical pH sensor was prepared as a multilayered structure. The absorbance values obtained from the UV–Vis spectrophotometer showed an increase of film absorbance as layers were added (Figure 2). This is due to the increase of accumulated CI concentration within the film layers. The increase of absorbance indicated the increase in sensor sensitivity [17].

### 3.2. Effect of pH on the Response Time, Reproducibility, and Lifetime of the Pe/CI Optical pH Sensor

**Figure 3:**
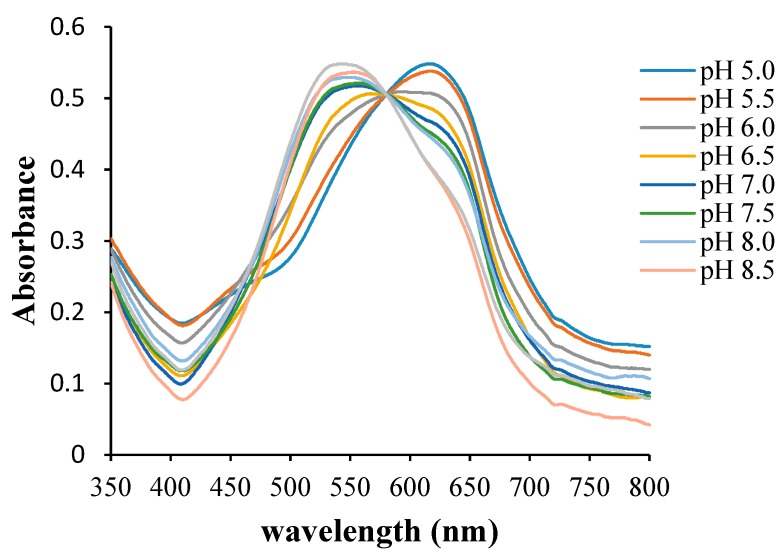
The response of the Pe/CI optical pH sensor to variations in pH in a PBS 10 mM solution, showing conversion from the protonated to the deprotonated form.

Any change in environmental pH can cause protonation or deprotonation on the sensor as a result of H+ ions interaction through mass transfer [18,19]. Therefore, the degree of pH change will determine protonation or deprotonation. The effects of pH on the Pe/CI optical pH sensor are shown in Figure 3. When the optical pH sensor was in an acidic environment, the maximum absorbance was obtained at the wavelength of 615 nm; this occurred when protonation took place. Meanwhile, when the optical pH sensor was in a basic environment, deprotonation occurred, and the maximum absorbance was observed at 535 nm. The process of transformation from protonation to deprotonation was indicated by an isosbestic point [20]. At this point, the film gave the optimum absorbance, either in acidic absorbance or in basic absorbance.

**Figure 4:**
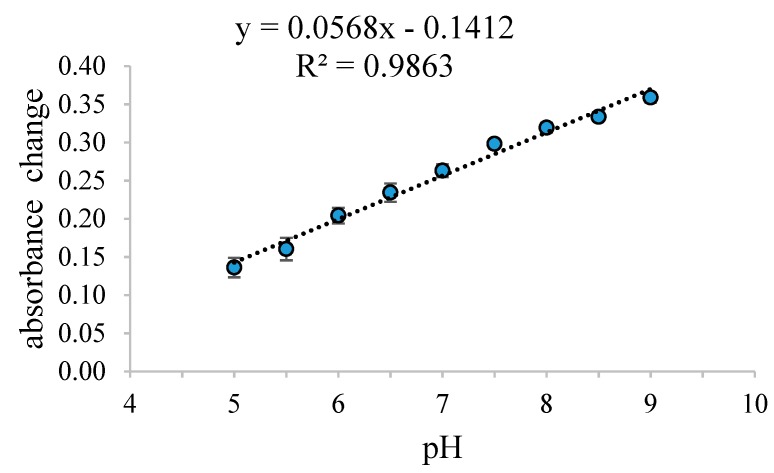
The response of the Pe/CI optical pH sensor in a pH range of 5–9.

This sensor exhibited excellent linearity (R^2^ = 0.97888) for the determination of pH in the range of pH 5–9 (Figure 4). This indicates that the Pe/CI sensor can be applied for accurate fish freshness monitoring at the pH range of 5–9. This linear pH range is comparable to that established by Bakker et al. [19], who also used a PVC/CI sensor. Another study using methacrylate acrylic/CI also exhibited a similar linearity for pH detection between 5.5 and 8.0 [21].

**Figure 5:**
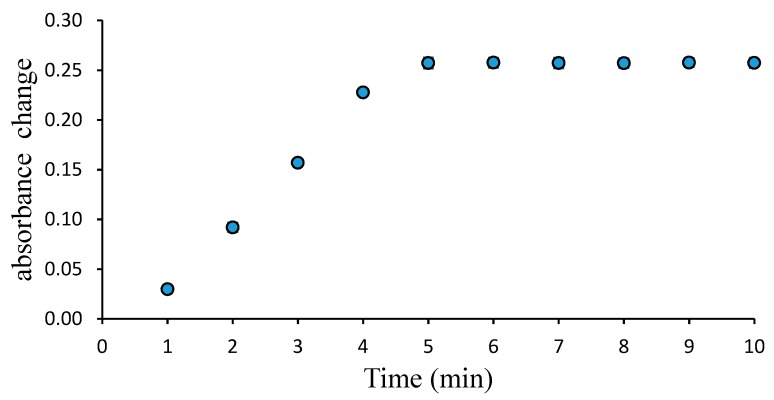
Pe/CI optical pH sensor response determined from 0.5 to 10 min.

The evaluation of the sensor’s response time was studied at 0.5–10 min. The results showed that sensor response stability was achieved at the 4th minute (Figure 5). This indicates that the Pe/CI optical pH sensor gives rapid response. A previous study using plasticizer-free acrylate/CI gave a slower response time of 10 min, due to the leisurely transport of CI within the polymeric film [21]. For polymers using plasticizers, such as PVC, the response time was shown to be less than 1 min. The mechanism of ion transport in the hydrogel optical pH sensor is incomparable to that of PVC plastic. For example, CI diffusion in a PVC plastic film is influenced by the polymeric ratio and by the types of plasticizer used. Therefore, the plasticizer content will increase the diffusion coefficient [22]. In the case of the hydrogel optical pH sensor, the response time is affected by the film density, which will, in turn, affect the proton transport process. Therefore, by optimizing the film density, a more rapid response time can be achieved.

The reproducibility of the Pe/CI optical pH sensor was determined by reproducing 10 sensors, and the relative standard deviation (%RSD) obtained was 1.49% (Table 1). The standard deviation value of <10% indicated that reproducibility was very good.

**Figure 6:**
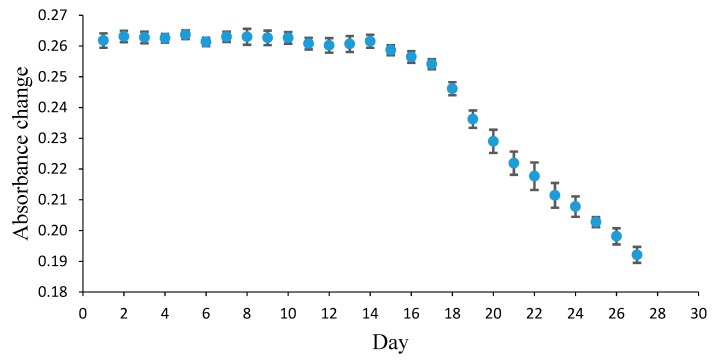
The response of the Pe/CI optical pH sensor determined on a day scale in a range of 1–27 days.

The sensor’s lifetime was also evaluated by determining the sensor’s response from 1 to 27 days (Figure 6). The optical pH sensor’s activity remained stable for almost two weeks and began to decline on the 15th day. An 8.6% decrease in response was observed between the 15th and the 20th day, followed by a decrease of 25% between the 20th and the 27th day. This showed that the optical pH sensor had good stability and could detect fish freshness within 20 days.

### 3.3. The Determination of Fish Freshness Using the Pe/CI Optical pH Sensor

The determination of fish freshness was carried out by evaluating the change in the pH of the fish, starting from fresh (alive) condition (Day 0) and continue during storage at 4 °C. The determination was conducted every day for 17 days.

**Figure 7:**
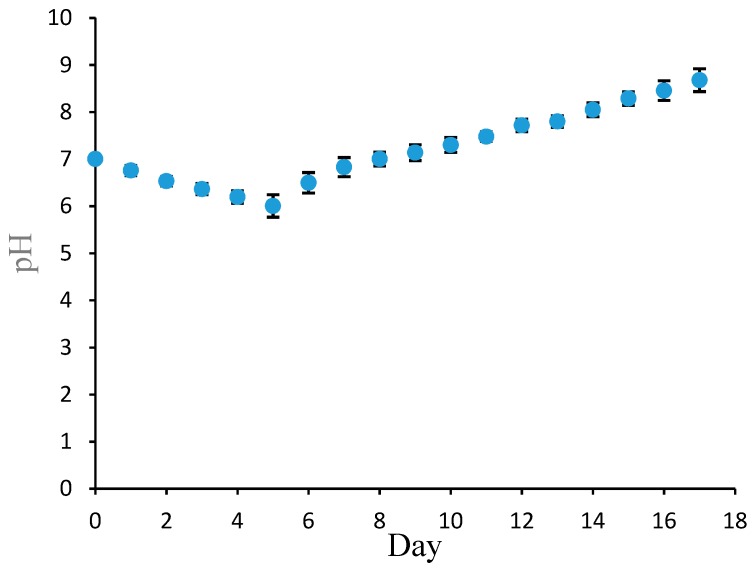
The response of the Pe/CI optical pH sensor on the fresh fish sample from day 0 to day 17.

The pH determination of the fish sample is shown in Figure 7. In fresh condition (0-day storage), the pH was determined to be 7. This is because the fish was in a pre-rigor phase, and the pH was neutral. After that, the fish pH decreased from 7 to 6 between day 1 and day 5. The decrease in pH indicated that the fish had entered rigor mortis. This phenomenon is caused by the degradation of glycogen, which results in lactic acid formation and causes the fish’s acidity to increase [23]. Enzyme activity is very slow at temperatures up to 17.8 °C [24]. The pH determination on day 7–20 exhibited a steady increase to pH 8.67. The increasing pH is the result of microbial enzymatic activity degrading the fish proteins and lipids, resulting in a basic condition [25]. This post-rigor mortis stage showed that the fish was not in good condition. These results show that the Pe/CI optical pH sensor exhibits excellent performance and can be applied to monitor fish freshness.

## 4. Conclusions

A pH optical sensor using a pectin membrane and a CI matrix was successfully constructed and characterized. The characterization of this sensor proved that it gives a fast response and produces desirable linearity and reproducibility. The sensor’s performance was tested on fish for the determination of freshness, and the sensor provided outstanding results, verifying that it can be successfully used on real samples.

## Figures and Tables

**Table 1:** Reproducibility of the Pe/CI optical pH sensor determined by reproducing 10 sensors.

Number of Sensors	Absorbance before Adding Buffer Solution	Absorbance after Adding PBS pH 7	Absorbance Change
1	0.206	0.446	0.240
2	0.204	0.441	0.237
3	0.208	0.454	0.246
4	0.205	0.451	0.246
5	0.204	0.447	0.243
6	0.205	0.451	0.246
7	0.203	0.448	0.245
8	0.204	0.444	0.240
9	0.201	0.45	0.249
10	0.202	0.447	0.245
Average			0.2437
STDV			0.003653
RSD (%)			1.498976
